# Genomic Prevalence of Heterochromatic H3K9me2 and Transcription Do Not Discriminate Pluripotent from Terminally Differentiated Cells

**DOI:** 10.1371/journal.pgen.1002090

**Published:** 2011-06-02

**Authors:** Florian Lienert, Fabio Mohn, Vijay K. Tiwari, Tuncay Baubec, Tim C. Roloff, Dimos Gaidatzis, Michael B. Stadler, Dirk Schübeler

**Affiliations:** Friedrich Miescher Institute for Biomedical Research, Basel, Switzerland; The Babraham Institute, United Kingdom

## Abstract

Cellular differentiation entails reprogramming of the transcriptome from a pluripotent to a unipotent fate. This process was suggested to coincide with a global increase of repressive heterochromatin, which results in a reduction of transcriptional plasticity and potential. Here we report the dynamics of the transcriptome and an abundant heterochromatic histone modification, dimethylation of histone H3 at lysine 9 (H3K9me2), during neuronal differentiation of embryonic stem cells. In contrast to the prevailing model, we find H3K9me2 to occupy over 50% of chromosomal regions already in stem cells. Marked are most genomic regions that are devoid of transcription and a subgroup of histone modifications. Importantly, no global increase occurs during differentiation, but discrete local changes of H3K9me2 particularly at genic regions can be detected. Mirroring the cell fate change, many genes show altered expression upon differentiation. Quantitative sequencing of transcripts demonstrates however that the total number of active genes is equal between stem cells and several tested differentiated cell types. Together, these findings reveal high prevalence of a heterochromatic mark in stem cells and challenge the model of low abundance of epigenetic repression and resulting global basal level transcription in stem cells. This suggests that cellular differentiation entails local rather than global changes in epigenetic repression and transcriptional activity.

## Author Summary

Epigenetic modifications of DNA and bound histones are major determinants of cell type–specific gene expression patterns. A prevalent model in stem cell biology suggests that the loss of pluripotency entails global increase in heterochromatin and coinciding shutdown of lineage unrelated genes. We performed analysis of both H3K9 dimethylation pattern and the global transcriptome in an advanced murine neuronal differentiation model. In this paradigm, we do not find evidence for a global increase in heterochromatic H3K9 dimethylation or reduction of transcriptome complexity as stem cells become terminally differentiated post-mitotic neurons. This suggests that pluripotent embryonic stem cells are not *per se* unique in regards to heterochromatin abundance and transcriptional plasticity as compared to somatic cells. Instead, focal changes in chromatin might help to stabilize cellular states at any developmental stage.

## Introduction

Resetting of the transcriptional program is the key driver for cell type specification during organismal development [1], [2]. While embryonic stem (ES) cells bear the fascinating ability to acquire very diverse fates, derived somatic stages are usually irreversible under physiological conditions. This unidirectionality has been suggested to depend in part on epigenetic repression of lineage unrelated genes [3], [4]. Accordingly, ES cell plasticity was suggested to rely on a low prevalence of heterochromatin and coinciding promiscuous low-level expression of many genes in stem cells [5]–[11]. In line with this model, distinct changes in nuclear staining had previously been observed by electron microscopy during cellular differentiation [12], [13]. Further, a subset of promoters was shown to become DNA methylated [14]–[16] and the repressive histone modifications H3K27me3 and H3K9me3 were reported to locally expand in differentiated cells [9].

Here, we set out to test the model of widespread heterochromatinization via monitoring of the differentiation-coupled dynamics of H3K9me2, a repressive epigenetic modification, which appears to be the most abundant heterochromatic modification and has recently been reported to cover large domains in differentiated cells [17]. Unexpectedly, we found that H3K9me2 is not only highly abundant in terminally differentiated cells, but already occupies large parts of the genome in pluripotent stem cells. In this cellular state, H3K9me2 occupies most genomic regions devoid of transcription and certain histone modifications. While our analysis revealed discrete local changes particularly at gene bodies, we observed little global increase in H3K9me2 during differentiation. This unexpected finding motivated us to revisit the model of promiscuous low-level gene expression in undifferentiated cells by quantitative RNA sequencing. Remarkably, we found the actual number of low-level expressed genes, postulated hallmarks of stem cells to be equal between both developmental states. Together, our findings challenge the model of promiscuous basal gene expression as a distinct property of pluripotency and a widespread increase of heterochromatin during cellular differentiation.

## Results

### H3K9me2 is nearly invariant and only displays distinct local changes between developmental stages

**Figure 1:**
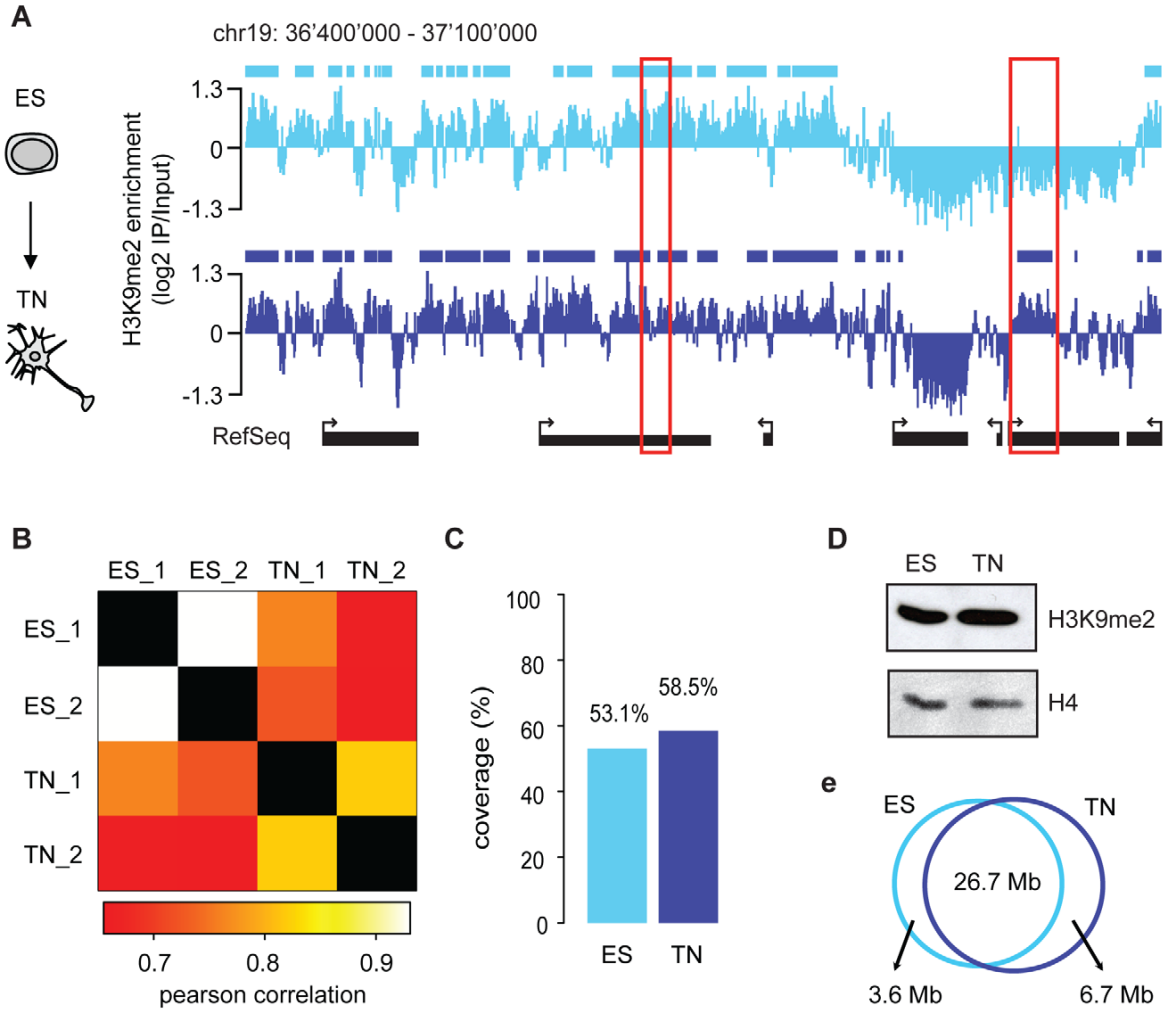
H3K9me2 covers large domains in pluripotent stem cells and derived neurons and is largely invariant between both states. (A) H3K9me2 localization in ES cells and neurons at a representative chromosomal region. Bars above each track indicate H3K9me2 domains determined by HMM. The red boxes highlight two regions that lose and gain H3K9me2, respectively. (B) Pair-wise correlation of H3K9me2 signal among different samples and experiments. Biological replicates are indicated as 1 and 2. Pearson correlations of IP/Input ratios were calculated for 500 bp windows on chromosome 19 (white/yellow corresponds to higher correlations). (C) Quantification of genomic coverage of H3K9me2 in ES cells and neurons applying a two-state HMM (i.e. “high” or “low”). Shown is the percentage of chromosome 19 that is in an H3K9me2 “high” state (i.e. enriched for H3K9me2) in both biological replicates. The value was normalized to the total coverage of the tiled region on the array. (D) Western blot detection of H3K9me2 levels in ES cells and neurons (TN). (E) Venn diagram showing the overlap of H3K9me2 enriched regions between ES cells and neurons (TN).

**Figure 2:**
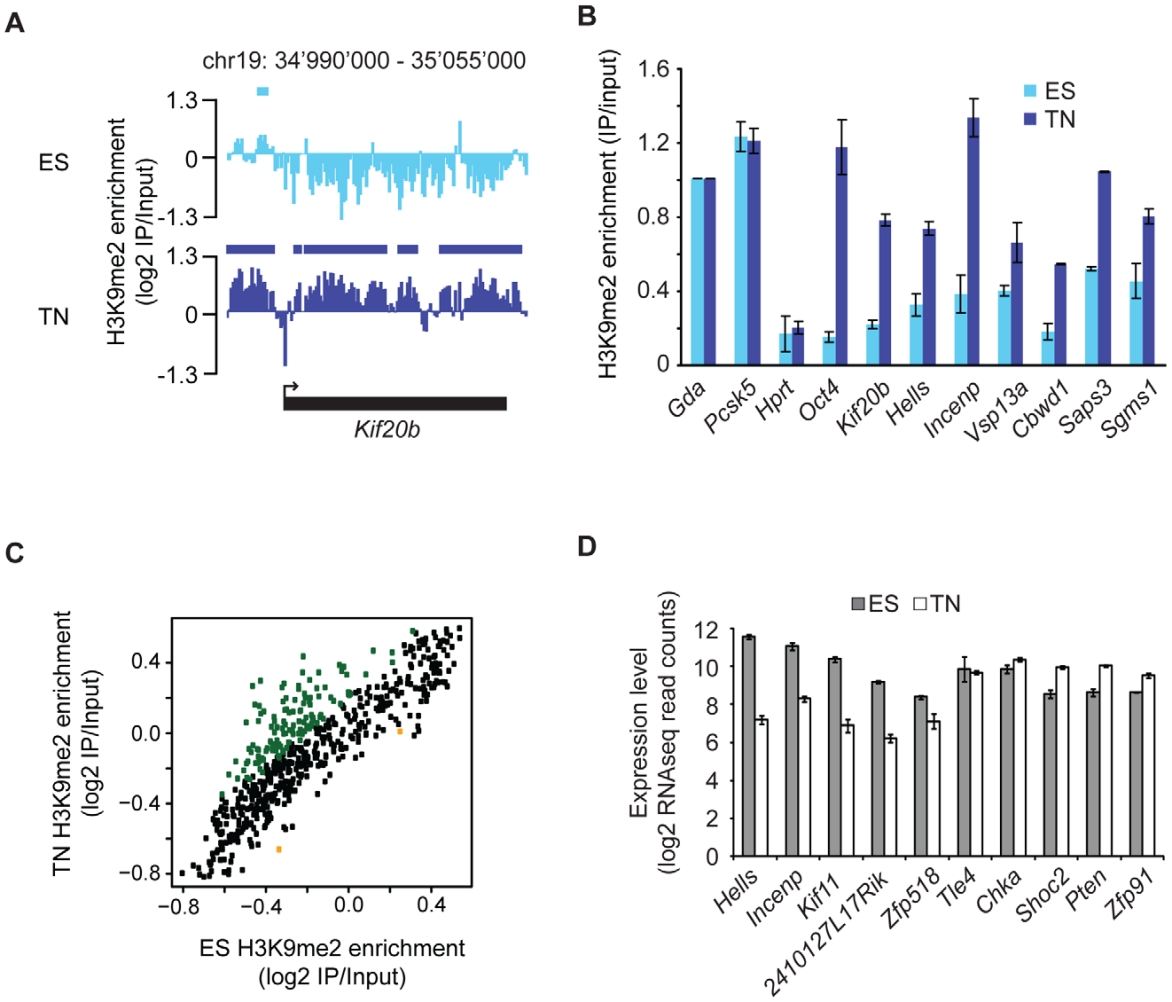
Changes in H3K9me2 during differentiation occur localized and at gene bodies. (A) H3K9me2 signal at the *Kif20b* locus as an example of a locus, which gains H3K9me2 in neurons. Bars above each track indicate HMM-defined H3K9me2 domains. (B) Validation of microarray results by single gene PCRs for a set of gene bodies that gain H3K9me2. Enrichments were normalized to *Gda*. Both *Gda* and *Pcsk5* have equally high H3K9me2 levels in both cellular states in the array data. Methylation levels of *Hprt* (negative control) and *Oct4* (*Pou5f1;* positive control [39]) were measured at the promoter. Shown are averages from 2 independent differentiation experiments. Error bars indicate standard deviation. (C) Comparison of H3K9me2 enrichments in gene bodies in ES cells and neurons. Genes, which gain and lose H3K9me2 significantly (adjusted P-Value <0.05) are depicted in green and orange, respectively. (D) RNAseq read counts of 10 genes that show the most significant gain of H3K9me2. Black and white bars indicate RNA in ES cells (ES) and neurons (TN), respectively.

To asses differentiation associated dynamics of the repressive histone modification H3K9me2 we made use of a highly pure and robust murine *in vitro* neurogenesis model [18], which we previously used to profile histone and DNA methylation [14]. Here, we generated profiles for H3K9me2 in pluripotent embryonic stem cells and derived terminally differentiated pyramidal neurons. We made use of custom tiling arrays covering 10% of the mouse genome including all well-annotated promoters, several large multi-gene loci and the complete chromosome 19 (see Figure S1 and Text S1). The chromosomal profiles for H3K9me2 revealed domains of enrichments that upon visual inspection were highly comparable between stem cells and the neuronal state (Figure 1A), which is further supported by a high overall pair-wise correlation (Figure 1B). Despite this overall similarity we noticed confined regional differences (Figure 1A), a finding which is consistent with the fact that biological replicates of H3K9me2 are more similar than the patterns between cell states (Figure 1B). We also included in our comparison a recently published dataset for H3K9me2 in a distinct ES cell line [17], which shows high correlation to our ES cell datasets despite different experimental conditions (Figure S2). Of note, analysis of the H3K9me2 dataset from Wen et al. [17] revealed that chromosome 19 behaves similar to the other chromosomes (Figure S3), suggesting that our results can be extrapolated to the entire genome. Together this demonstrates that our H3K9me2 data are reproducible and of high resolution, yet overall patterns appear to be highly similar between a pluripotent and a terminally differentiated state. Visual inspection suggests that H3K9me2 covers large domains in both ES cells and neurons (Figure 1A). To quantitatively define the actual location and sizes of domains we applied a Hidden-Markov-Model (HMM) analysis to the microarray data. This unsupervised statistical method is a widely accepted approach for unbiased data segmentation in epigenome analysis [19], [20]. The HMM analysis not only agreed with and statistically corroborated the visual impression of the raw data, but also yielded robust results under variable settings (Figure S4). It revealed that over 50% of chromosome 19 is covered by H3K9me2 in ES cells (Figure 1C). Using the same approach for the H3K9me2 data in the ES cell-derived terminally differentiated neurons, we detected a modest yet reproducible 5% increase of genomic regions covered by H3K9me2 (Figure 1C). We conclude that global coverage and size of H3K9me2 domains is nearly identical between ES cells and derived post-mitotic pyramidal neurons. In line with this finding we do not detect a significant change in global H3K9me2 levels by Western blot detection (Figure 1D). Moreover, H3K9me2 domain features of ES cells and neurons show similar size distribution and median length (Figure S4). Further analysis revealed that changes in H3K9me2 between the two examined cellular states are rare; 88% of H3K9me2 occupied regions in ES cells are also occupied in neurons (Figure 1F). Notably, regions that change in H3K9me2 state tend to be small and are below the average size of invariant domains (Figure S5). Consistent with the overall increase of 5% in H3K9me2 coverage during differentiation, regions which gain H3K9me2 are more frequent and of larger size than regions showing a loss of the mark (Figure S5 and Figure S6). Interestingly, most of the larger regions (>10 kb) that gain H3K9me2 are located within genes, starting downstream of the promoter region (Figure 2A and Figure S5). These global findings are fully reproducible in single gene controls (Figure 2B) and consistent with a focused comparison of only genic regions (Figure 2C). Importantly, this shows that our experimental and data analysis approach is indeed highly sensitive to detect differences if they do occur. Interestingly, many genes that acquire H3K9me2 show slightly reduced expression in many cases, while others increase expression upon gain of the modification (Figure 2D and Figure S5). This suggests that the gain of H3K9me2, while highly selective for gene bodies, cannot simply be explained by the silencing of gene activity.

### H3K9me2 and H3K27me3 are mutually exclusive

**Figure 3:**
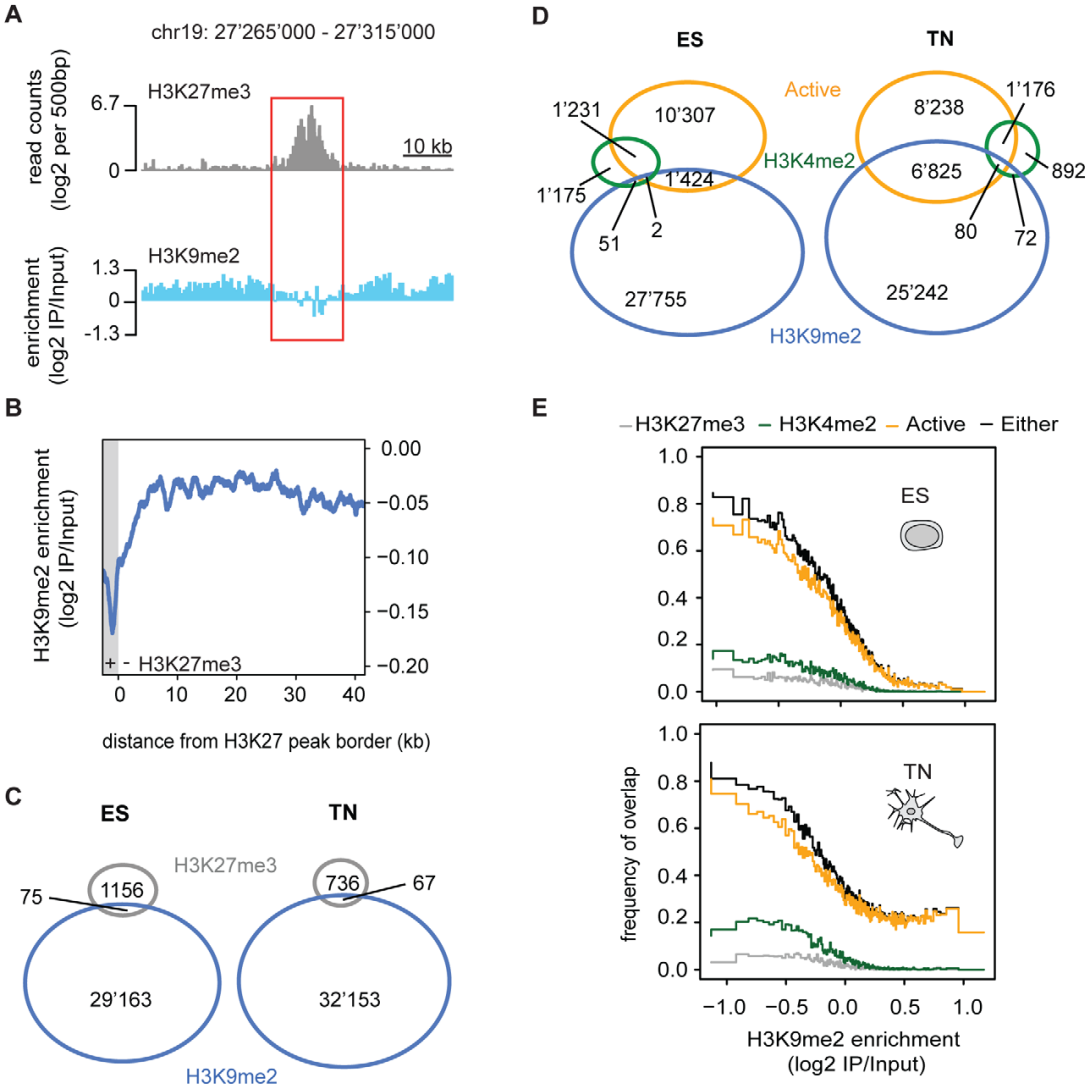
H3K9me2 occurs mutually exclusive with expression and distinct chromatin marks. (A) Enrichment for H3K27me3 and H3K9me2 in a representative region. (B) H3K9me2 enrichments relative to peaks of H3K27me3 illustrating the sharp boundaries between both marks. Plotted are the moving-window averages (median) of H3K9me2 enrichments with window sizes of 500 bp (blue line) around all H3K27me3 domains on chromosome 19 (shown in grey). (C) and (D) Venn diagram illustrating the actual overlap between H3K9me2 and H3K27me3 occupied regions (C) and between H3K9me2, H3K4me2 occupied and active regions (D). Numbers indicate base pair coverage on chromosome 19 (in kb). Note that areas are not drawn to scale. (E) The higher H3K9me2, the less likely a genomic region is active or contains H3K27me3 or H3K4me2. H3K9me2 enrichment values of 500 bp windows were binned into groups of equal size. Plotted is the frequency for H3K9me2 enrichment bins to overlap with active regions (orange), H3K27me3 occupied (gray), H3K4me2 occupied (green) and regions in either of these states (black).

Given the high prevalence of H3K9me2, we next asked how its presence relates to a distinct repressive chromatin modification, namely trimethylation of H3K27 (H3K27me3). This mark is set by the Polycomb pathway and often occurs in domains of several kilobases [9], [21], [22]. We find that both heterochromatic histone modifications occur mutually exclusive even when in direct neighborhood as illustrated by the sharp boundaries of the H3K9me2 signal next to H3K27me3 peaks (Figure 3A, 3B and 3C). This is consistent with a previous study in human embryonal carcinoma cells that was limited to promoters [23].

### H3K9me2 is largely exclusive with active chromatin

We further related H3K9me2 occupancy to regions with transcriptional activity or presence of the active modification H3K4me2. Active regions are mutually exclusive with H3K9me2 in ES cells but surprisingly to a lesser extent in neurons (Figure 3D). The compatibility of H3K9me2 and gene expression in neurons is however limited to gene bodies and does not occur in the promoters of expressed genes, consistent with the former regions gaining H3K9me2 during differentiation (Figure 2A and Figure S7). We find the majority of H3K4me2 regions to be mutually exclusive with H3K9me2 in stem cells (Figure 3D). In neurons, a small number of regions become co-occupied, again most of these being within transcribed genes (Figure 3D). Importantly, an HMM independent analysis confirms that regions with high H3K9me2 enrichment do not overlap with transcribed genes in stem cells, yet a subset does in neurons (Figure 3E). We conclude that gain of H3K9me2 during differentiation has only a minor effect on the overall chromosomal coverage of the modification, yet it occurs highly localized and preferentially at genic regions.

### Prevalent low-level transcription is not a stem cell–specific feature

**Figure 4:**
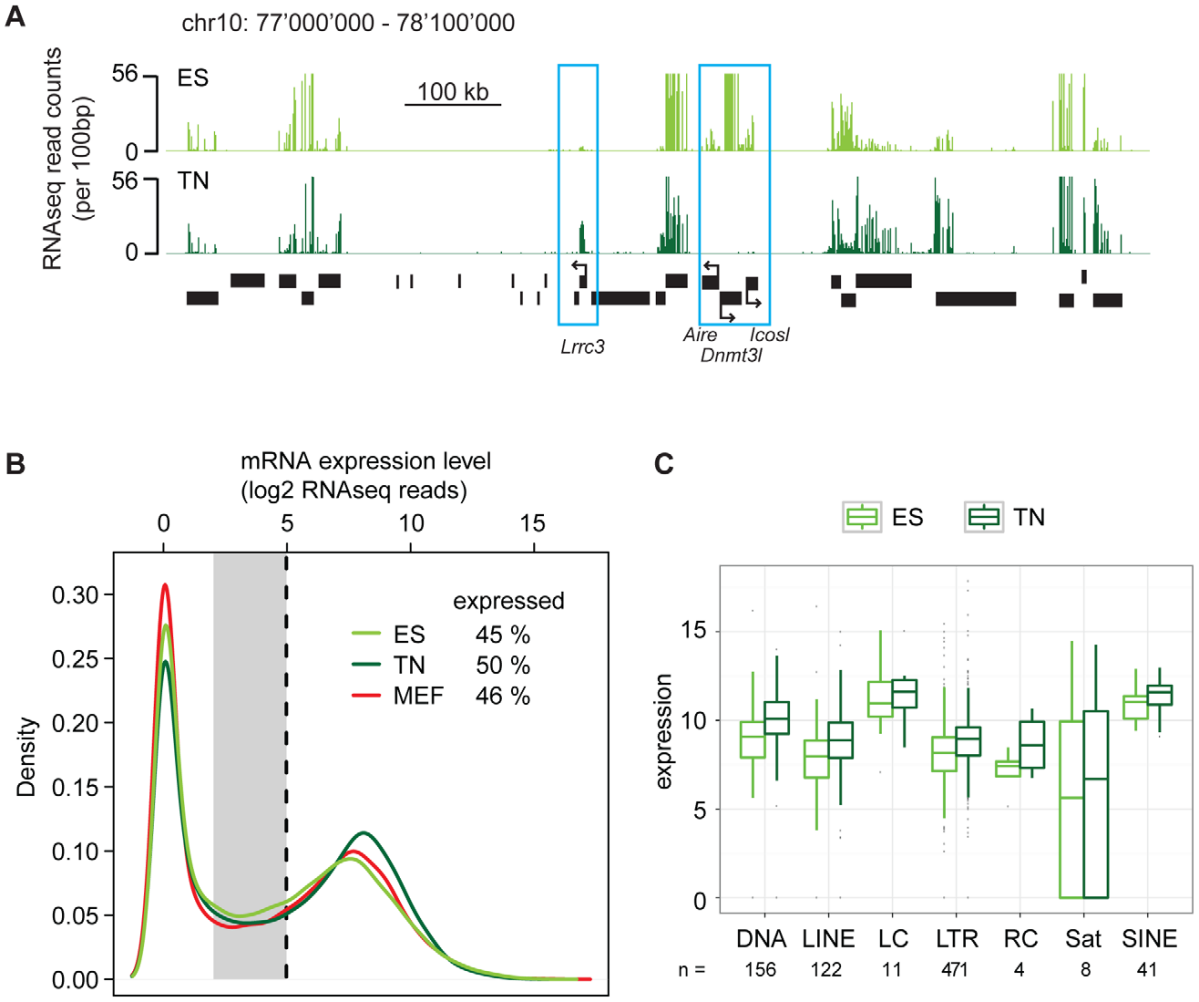
Promiscuous low-level expression is not a distinctive feature of the pluripotent state. (A) Representative region illustrating RNAseq data in ES cells and neurons (TN). Blue boxes indicate genes that either increase (*Lrrc3*, a neuron specific ion channel) or decrease (*Aire*, *Dnmt3l*, *Icosl*; three ES cell specific genes) expression during differentiation, while the other genes remain constant. (B) Density distribution of transcript abundance (measured as read counts) for all RefSeq transcripts in ES cells (green line), derived neurons (dark green line) and mouse embryonic fibroblasts (red line). Numbers indicate percentage of expressed genes in each cell type based on a threshold of log2(read count) = 5 (black dotted line). The gray area between 2 and 5 indicates the zone of low-level expression. (C) Boxplot representation of expression levels of prominent repeat classes in ES cells and neurons (TN). Numbers below indicate how many repeat elements are included in each repeat class.

Our finding of surprising conservation of heterochromatin patterns in a refined model of differentiation let us to revisit the transcriptome in a quantitative manner using high throughput RNA sequencing (RNAseq). RNAseq in ES cells and derived neurons revealed the expected down regulation of stem cell specific genes and induction of neuron specific genes (Figure 4A). Further, when counting RNA molecules after gene mapping and normalization, both cell types displayed a characteristic bimodal distribution. This reflects a group of genes that is in a clear off state with no detectable RNA molecules and a second peak of expressed genes (Figure 4B). Separating these two groups of genes by a stringent cutoff revealed that out of 35′606 transcription units, 45% are expressed in ES cells. Interestingly, we identified a slightly higher number (50%) of expressed genes in terminally differentiated neurons, indicating that differentiation of stem cells is not coinciding with a reduced number of highly expressed genes. This agrees with a recent report that suggested that stem cells and somatic cells do mainly differ in the number of low-level expressed genes due to a global reduction of basal gene activity in the course of lineage-commitment and loss of pluripotency [10]. To test this in our *in vitro* differentiation system, we grouped the genes that could not clearly be assigned to the on or off state, into a separate class of genes expressed at low to background level (Figure 4B). This analysis reveals that in stem cells 16% of all transcription units show a basal expression level. Surprisingly however, the proportion of genes expressed at such low level (14%) is very similar in neurons. This unexpected finding prompted us to conduct an additional RNAseq experiment in a second fully differentiated somatic murine cell type; primary mouse embryonic fibroblasts (MEFs). Interestingly, also fibroblasts display a similar transcriptional landscape as stem cells, with 46% of all transcription units being highly expressed and 13% being expressed at basal levels. Hence, this qualitative similarity of expression patterns is not specific to the neuronal subtype we generated *in vitro*, but appears to be a more general property of both undifferentiated and differentiated cells. Thus, while transcripts expressed at low levels show little overlap between stem cells and somatic cells (Figure S8), their numbers are remarkably similar. Stem cells do also not show an increased number of highly expressed genes. Based on additional analysis we can exclude that this similarity of the transcriptional landscape is a consequence of insufficient sampling (Figure S9). Moreover, it is not limited to genic regions as the abundance of transcripts generated from diverse classes of endogenous repeat is comparable between stem cells and neurons (Figure 4C).

## Discussion

Embryonic stem cells are characterized by their potential to differentiate into any cell type of the three germ layers in the developing embryo, while somatic cells lose this developmental plasticity upon lineage-commitment. Despite its relevance for our understanding of development and disease, the molecular determinants of pluripotency are still not fully understood and the factors responsible for this uniqueness of stem cells are actively debated [24]. Our study of gene expression and an abundant heterochromatin mark reveal surprising conservation of the transcriptome and epigenome landscape between pluripotent and unipotent cells. H3K9me2 is already highly prevalent in ES cells, arguing that the pathways that mediate H3K9me2 are highly active in stem cells, and serve similar functions as in somatic cells, which only show a slight increase of the mark (from 53 to 58%). Interestingly, the observed gain occurs very localized at gene bodies and does not necessarily coincide with lower transcription of the corresponding gene. The analysis of regions that acquire H3K9me2 during differentiation further revealed a differentiation specific coexistence of H3K9me2 and transcriptional activity, which is not detected in the pluripotent state and which could be involved in modulating expression in the differentiated cell. Nevertheless, despite the subtle increase we detected, an involvement of H3K9me2 in globally regulating cell-type specific gene repression appears unlikely. The limited dynamics as compared to massive transcriptome reprogramming and the limited correlation between expression and gain of H3K9me2 at target genes argue against H3K9me2 as being a major player in setting up gene expression programs.

These findings disagree with a recent report that suggested absence of large H3K9me2 domains in ES cells and found a striking increase in differentiated cells [17]. Notably, our ES cell profile for H3K9me2 is similar to the one generated previously (Figure 1B and Figure S2), making data analysis a likely explanation for the discrepancy. We applied an unbiased approach that is insensitive to variations between arrays and which we show to lead to similar results under various parameter settings (Figure 3 and Figure S10). As already discussed by Fillion and van Steensel [25], the previous study relied on defined thresholds, which can be prone to false estimation of differences particular in the absence of biological replicates [25].

Widespread low-level expression in stem cells has previously been reported and interpreted as a sign of pluripotency [5], [6], [10]. It has been speculated that this basal promiscuous activity would poise genes for rapid induction upon receipt of differentiation cues [5], [6], [10]. Using mRNA sequencing in our differentiation paradigm does not confirm this model. We do not find evidence of elevated transcription throughout the genome or on specific chromosome (Figure S8; [10]). A likely explanation for these discrepancies is that microarrays, which were used in the previous studies, overestimate low level signal due to cross-hybridization [26]. In the present study we utilized RNAseq, which permits an actual counting of RNA molecules and thus enables accurate discrimination between very low and no expression. Notably, RNA sequencing experiments have recently put other findings in question that relied on quantifying small transcriptome differences detected by microarrays. For example, recent RNAseq data challenged the presence of pervasive intergenic transcription [26] and the existence of transcriptional dosage compensation of the single male X chromosome [27]. In addition to the increased sensitivity of RNAseq that can explain the differences to previous studies, the presence of a small fraction of differentiated cells in suboptimal conditions of stem cell culture could similarly contribute to an overestimation of the number of genes that are actually expressed in stem cells. Notably, the ES cell differentiation protocol applied by us is optimized to reduce the number of differentiated cells in the culture [18].

In summary, our analysis suggests to revisit the model of massive heterochromatinization during cellular differentiation via a global increase in repressive histone marks [5]–[9], [17] and coinciding repression of basal gene activity [5], [6], [10].

Our data together with previous reports on dynamics of DNA methylation, H3K9me3 and the Polycomb pathway between pluripotent and somatic cells [9], [14], [15], [28]–[31] support a model whereby repressive chromatin is already highly active in stem cells and that epigenome reprogramming entails localized changes of repressive histone modifications and DNA methylation at regulatory regions that specify and stabilize lineage specification and terminal differentiation [32]. It will be interesting to determine if these local differences account for the observed changes in nuclear morphology [33]. Notably, epigenetic repression can be overcome by the local activity of transcription factors upon strong induction cues during normal differentiation or artificially during generation of induced pluripotent stem cells (iPS) [34] and might therefore safeguard rather than actively channel development via direct transcriptome regulation.

## Methods

### Cell culture

Wild-type embryonic stem cells (129Sv-C57Bl/6) were cultured and differentiated as previously described [14], [35]. Fibroblasts were isolated from wild-type embryos (C57Bl/6).

### Western blot and peptide dot blot analysis

Peptide sequences can be found in Table S2. Western blot analysis was performed with acid extracts using 1/1000 dilutions of either anti-H3K9me2 (Abcam no. 1220) or anti-H4 (Upstate, no. 07–108) antibodies. Blots were developed with ECL reagent (GE Healthcare).

### Chromatin-IP (ChIP)

ChIP experiments were performed as described before [14], starting with 70 µg of chromatin and 5 µg of the following antibodies: anti-dimethyl-H3K9 (LP Bio, no. AR-0108), anti-dimethyl-H3K9 (Abcam no. 1220), anti-trimethyl-H3K27 (Upstate, no. 07–449), anti-dimethyl-H3K4 (Upstate, no. 07–030). H3K9me2 ChIP samples were amplified using the WGA2 kit (Sigma) and hybridized to a custom tiling microarray (NimbleGen Systems Inc., see below). H3K27me3 and H3K4me2 ChIP libraries for Illumina sequencing were prepared with the Illumina ChIP-Seq DNA Sample Prep Kit (Cat# IP-102-1001) according to Illumina's instructions and sequenced on the Genome Analyzer II following the manufacturer's protocols. ChIP-real time PCR was performed using SYBR Green chemistry (ABI) and 1/40 of ChIP or 20 ng of input chromatin per PCR reaction. Primers are listed in Table S1.

### Microarray design

H3K9me2 ChIP samples were hybridized to custom designed microarrays representing all well-annotated promoters, several large multi-gene loci and the complete chromosome 19 with an average probe spacing of 100 bp and a total of 2.1 million features (HD2.1, NimbleGen Systems Inc).

### Microarray hybridization and analysis

Sample labeling, hybridization and array scanning were performed by NimbleGen Systems Inc. according to standard procedures. For analysis, raw fluorescent intensity values were used to calculate log2 of the bound/input ratios for each individual oligo. Subsequently, for comparison all arrays were normalized to a median log2 = 0 and scale normalized to have the same median absolute deviation using the “LIMMA” R/Bioconductor package [36], [37].

### RNAseq data analysis

RNA from ES cells, neurons and fibroblasts of two independent biological replicates each was used for cDNA preparation using oligo dT primers followed by sequencing on an Illumina GA II analyzer. Reads were mapped to the *Mus musculus* transcriptome and normalized to transcript length and sequencing library size (for details see Text S1).

### Bioinformatics

Unless otherwise stated, H3K9me2 enriched regions were identified by HMM and H3K4me2 and H3K27me3 peaks using MACS peak finder [38]. Active regions were defined as RefSeq transcription units with a normalized RNAseq log2 read count above 5 (for details see Text S1). Microarray design, hybridization and analysis, ChIPseq and RNAseq analysis and additional references are described in Text S1.

### Datasets

Microarray and deep sequencing data were deposited at NCBI's Gene Expression Omnibus and are accessible through GEO Series accession number GSE27866 (http://www.ncbi.nlm.nih.gov/geo/query/acc.cgi?acc=GSE27866).

## Supporting Information

Figure S1 Specificity of H3K9me2 antibodies used in this study. Indicated amounts of either modified or unmodified H3 and H4 peptides (H3 residues 1–20, 19–38 and 25–45, H4 residues 12–31) were spotted onto polyvinylidene difluoride membranes (GE) and probed with (A) anti-dimethyl-H3K9 (LP Bio, no. AR-0108) or (B) anti-dimethyl-H3K9 (Abcam no. 1220) at 1∶1000 dilution each. Both antibodies are highly specific towards H3K9me2.
(TIF)

Figure S2 High similarity of ES cell H3K9me2 datasets. (A) Comparison of our H3K9me2 enrichment profiles in ES cells to a previously generated ES cell profile (Wen et al., 2009). (B) Venn diagram illustrating the overlap of H3K9me2-occupied regions between the two ES cell datasets from (A) (defined by an HMM approach). The overlap agrees with the high correlation for this previously generated ES cell dataset with our two ES cell replicates (Pearson correlation of 0.73 and 0.74, on 500 bp windows).
(TIF)

Figure S3 H3K9me2 enrichments on chromosome 19 are representative of the entire genome. (A) H3K9me2 enrichments in ES cells from a previously generated dataset (Wen et al., 2009). The boxplots show H3K9me2 enrichment values for all probes separated by chromosome, with chromosome 19 highlighted in blue. (B) H3K9me2 enrichments per 900 bp promoter windows (defined in Mohn et al., 2008) in ES cells. The boxplots represent average enrichments at promoters separated by chromosome with chromosome 19 highlighted in blue.
(TIF)

Figure S4 H3K9me2 enriched regions are robustly identified independent of HMM parameters and H3K9me2 domain size is highly similar in ES cells and neurons. (A) Shown is the comparison of H3K9me2 enrichments in ES cells and neurons using a 3 state HMM to define non-occupied (low), intermediately and highly occupied domains (top). Importantly, the state specific HMM distribution parameters (mean and variance of H3K9me2 enrichment) are very similar between ES cells and neurons (bottom). Note that with a 3 state HMM the percentage of domains in the high state does not change between ES cells and neurons (TN). However, we detect an overall increase of the intermediate state of around 10%. (B) H3K9me2 domains were defined by HMM (see methods). While numbers of domains on chromosome 19 increase from 1618 to 1914 from ES cells to neurons, their domain size distribution is very similar. This was also seen using alternative domain definition methods (data not shown).
(TIF)

Figure S5 Regions that lose are smaller than regions that gain H3K9me2 during differentiation and gaining regions often overlap with gene bodies. (A and B) Histograms displaying the size distributions of regions that lose (A) and gain (B) H3K9me2 during neuronal differentiation. Adjacent 500 bp windows being in a low HMM state in both ES cell replicates and in a high HMM state in the neuron replicates were grouped into H3K9me2 losing and gaining regions, respectively. (C) Pie chart illustrating the percentage of large (>10kb) H3K9me2 gaining regions within (grey) and outside (white) of genes (left). This overlap is significantly higher than for all regions that have no H3K9me2 in ES cells, i.e. which are in a HMM low state in both replicates (right). (D) Boxplot showing expression levels of all genes (n = 138, adjusted P-value <0.05) that gain H3K9me2 in neurons. Note that the median expression does not change significantly between ES cells and neurons (TN), indicating that there is no global trend towards either up- or down-regulation upon accumulation of H3K9me2 in the gene bodies of these genes.
(TIF)

Figure S6 Regions losing H3K9me2 during differentiation are close to background. (A–C) Shown are representative genomic regions that significantly lose H3K9me2 as defined by HMM analysis. (D) ChIP-real time PCR validation of the regions shown in (A–C). Note that H3K9me2 enrichments are already low in ES and only slightly above background as exemplified by the active housekeeping gene *Hprt*, which does not carry H3K9me2. The loss of enrichment in neurons is small, though reproducible.
(TIF)

Figure S7 H3K9me2 in neurons rarely overlaps with promoter regions of active genes. (A and B) Pie chart illustrating the percentage of promoters (1 kb up- and downstream of TSS) (A) and gene bodies (B) of transcribed genes that overlap H3K9me2-occupied regions in neurons. Note that promoters are rarely H3K9 dimethylated when the gene is actively transcribed.
(TIF)

Figure S8 Overlap of low-level expressed genes and expression of transcripts per chromosome. (A) Venn diagram illustrating the overlap of transcripts expressed at a low level (log2 RNAseq reads between 2 and 5) in at least one of the examined cell types. Note that areas are not drawn to scale. (B) Boxplots showing expression levels of transcripts per chromosomes.
(TIF)

Figure S9 Absence of difference in transcriptome complexity is not a function of sample size in next generation sequencing. Shown is the number of detected Refseq transcripts (at least 32 reads) relative to the number of sampled reads. Number of detected transcripts is shown as the mean of 100 rounds of random subsampling with the same total read numbers. Irrespective of the sample size, a random sampling of RNAseq reads from ES cells (green line), neurons (dark green line) and MEFs (red line) revealed a smaller number of detected transcripts than an artificial sample with pooled reads from all three cell types (blue line) at any given sample size.
(TIF)

Figure S10 H3K9me2 domain definition by different statistical methods leads to similar results. (A) HMM-independent quantification of genomic coverage of H3K9me2 in ES cells and neurons. Shown is the percentage of chromosome 19 that lies within H3K9me2 peaks defined by a simple threshold method (t-peaks, see Text S1). (B) Diagram illustrating the actual overlap between regions defined to be occupied by H3K9me2 by two different methods; a simple threshold method (t-peaks; see Text S1) and by an HMM approach (HMM; see Text S1).
(TIF)

Table S1 List of real time PCR primers.
(TIF)

Table S2 Peptides used in dot blots in Figure S1.
(TIF)

Text S1 Supplementary methods.
(DOC)
