# Supplementary figures and images for: Genomic Prevalence of Heterochromatic H3K9me2 and Transcription Do Not Discriminate Pluripotent from Terminally Differentiated Cells
Source: PLoS Genet. 2011 Jun 2;7(6):e1002090. doi: 10.1371/journal.pgen.1002090 (PMC3107198; doi:10.1371/journal.pgen.1002090)

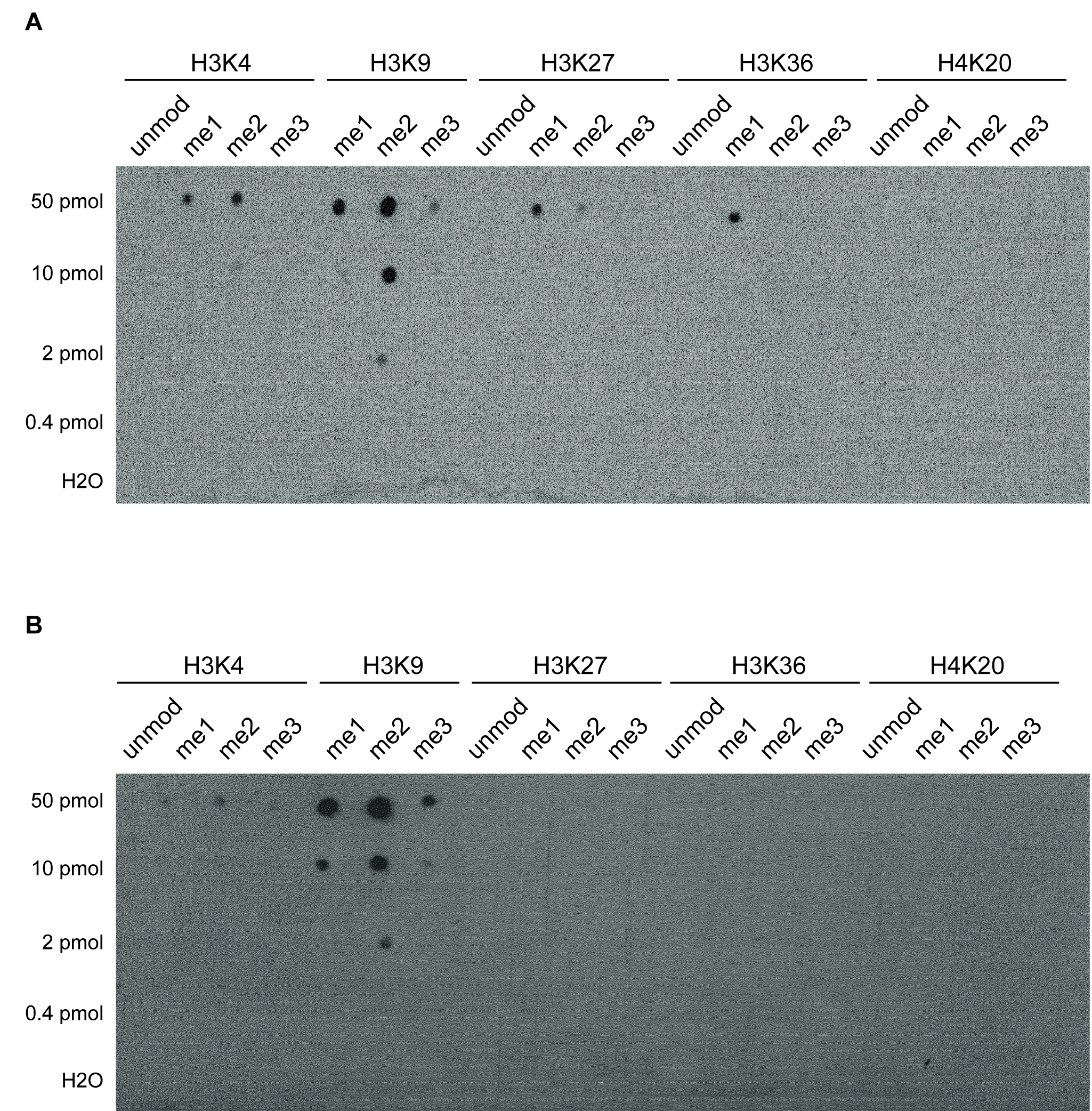

Supplement: Figure S1 — Specificity of H3K9me2 antibodies used in this study. Indicated amounts of either modified or unmodified H3 and H4 peptides (H3 residues 1–20, 19–38 and 25–45, H4 residues 12–31) were spotted onto polyvinylidene difluoride membranes (GE) and probed with (A) anti-dimethyl-H3K9 (LP Bio, no. AR-0108) or (B) anti-dimethyl-H3K9 (Abcam no. 1220) at 1∶1000 dilution each. Both antibodies are highly specific towards H3K9me2. (TIF) [file pgen.1002090.s001.tif]

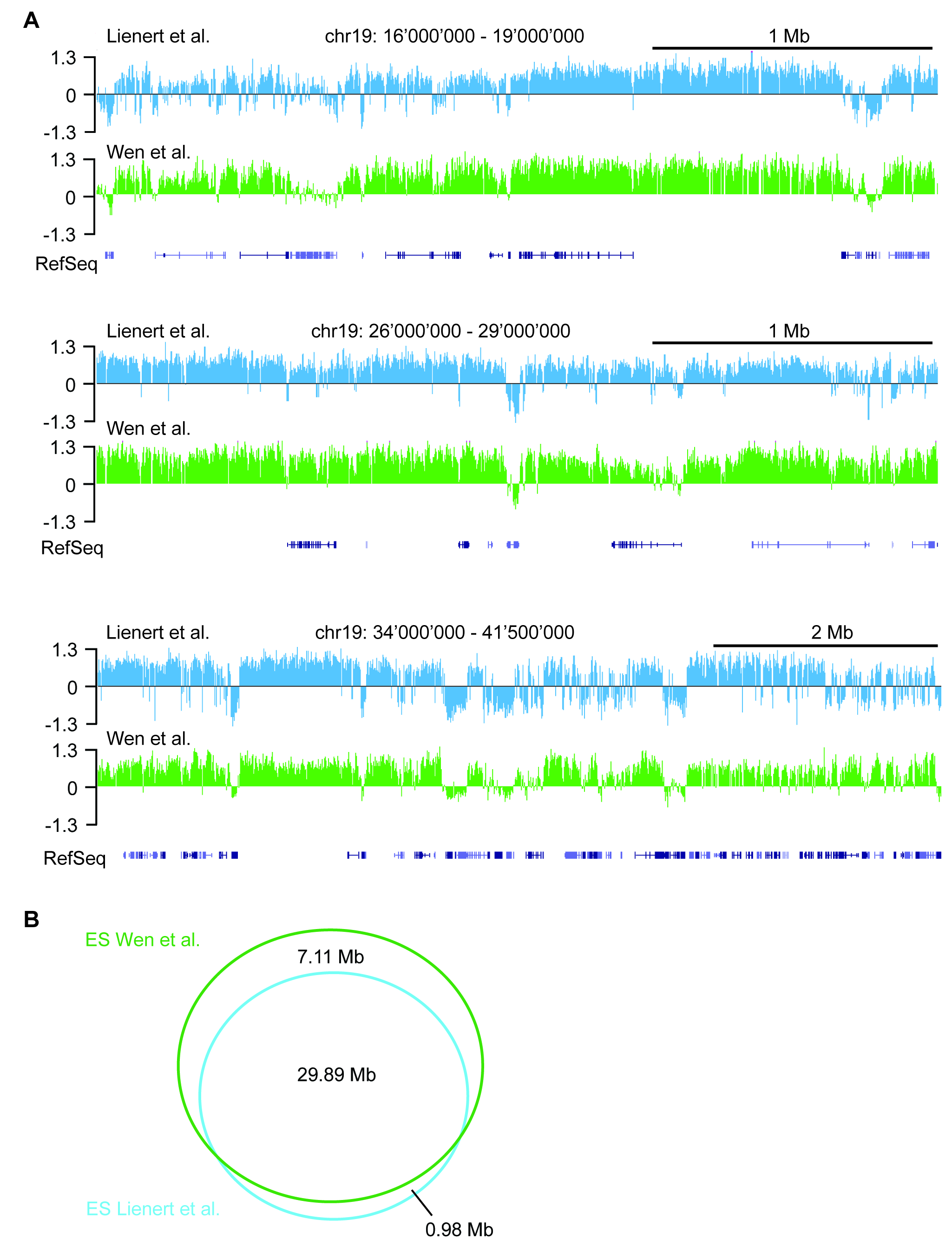

Supplement: Figure S2 — High similarity of ES cell H3K9me2 datasets. (A) Comparison of our H3K9me2 enrichment profiles in ES cells to a previously generated ES cell profile (Wen et al., 2009). (B) Venn diagram illustrating the overlap of H3K9me2-occupied regions between the two ES cell datasets from (A) (defined by an HMM approach). The overlap agrees with the high correlation for this previously generated ES cell dataset with our two ES cell replicates (Pearson correlation of 0.73 and 0.74, on 500 bp windows). (TIF) [file pgen.1002090.s002.tif]

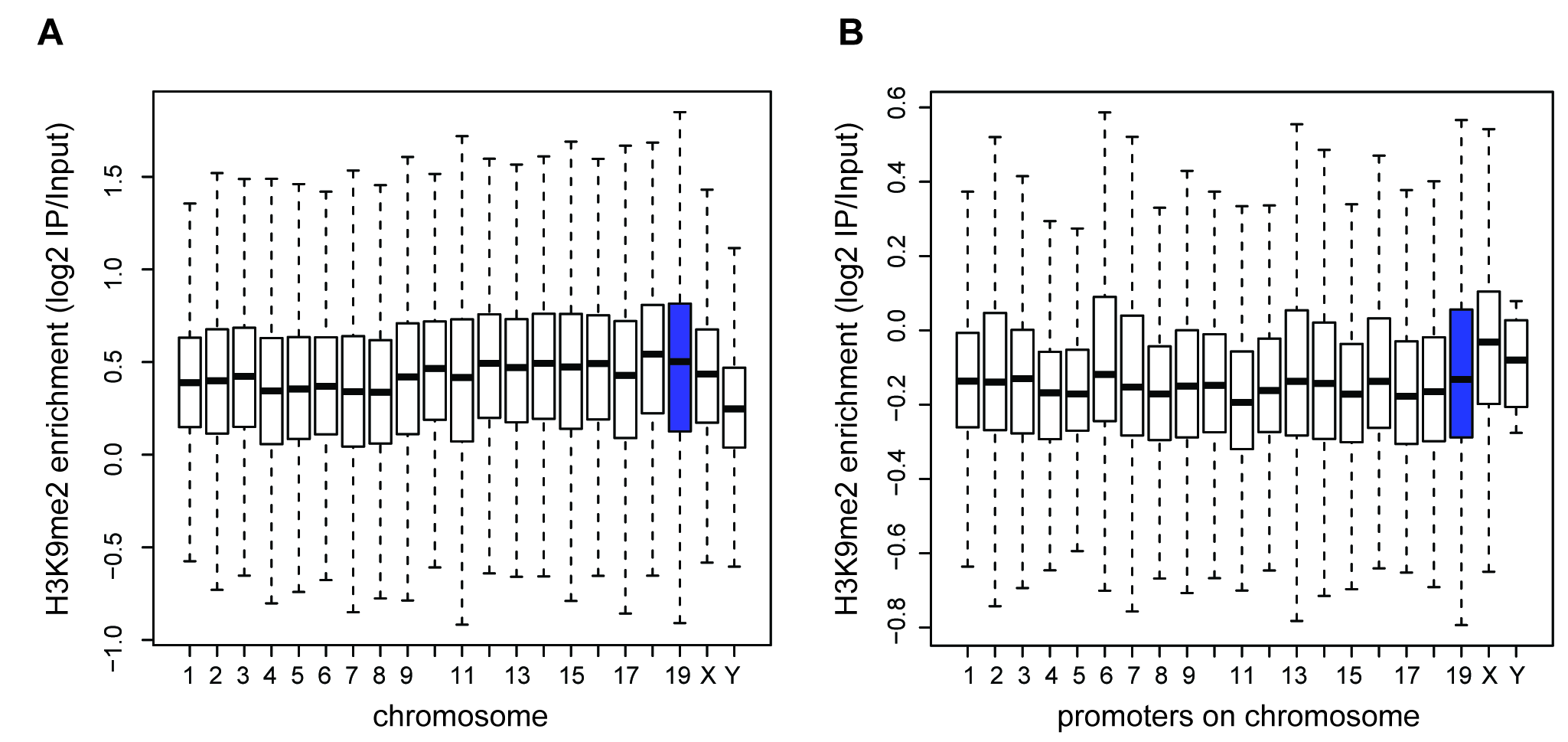

Supplement: Figure S3 — H3K9me2 enrichments on chromosome 19 are representative of the entire genome. (A) H3K9me2 enrichments in ES cells from a previously generated dataset (Wen et al., 2009). The boxplots show H3K9me2 enrichment values for all probes separated by chromosome, with chromosome 19 highlighted in blue. (B) H3K9me2 enrichments per 900 bp promoter windows (defined in Mohn et al., 2008) in ES cells. The boxplots represent average enrichments at promoters separated by chromosome with chromosome 19 highlighted in blue. (TIF) [file pgen.1002090.s003.tif]

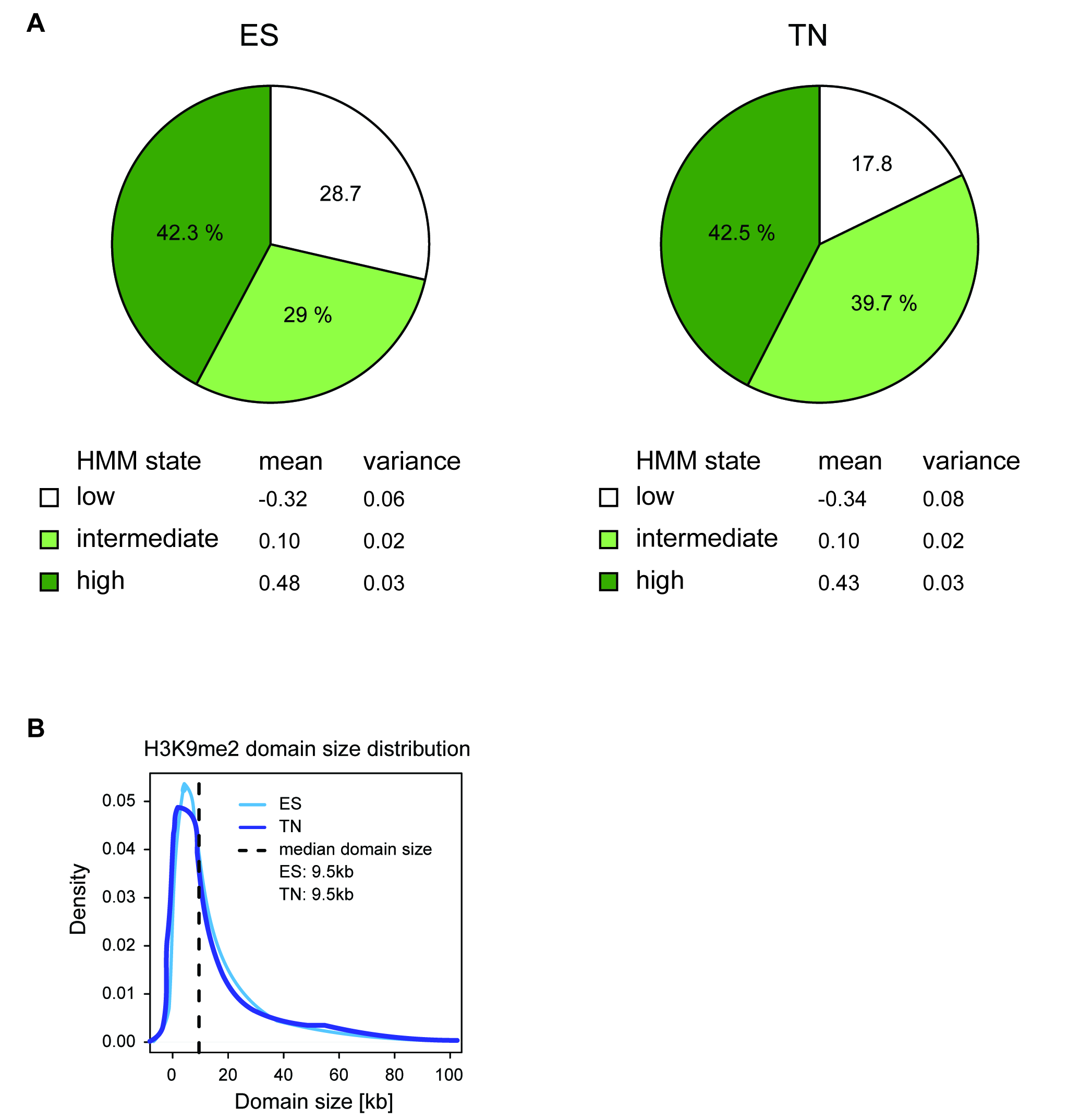

Supplement: Figure S4 — H3K9me2 enriched regions are robustly identified independent of HMM parameters and H3K9me2 domain size is highly similar in ES cells and neurons. (A) Shown is the comparison of H3K9me2 enrichments in ES cells and neurons using a 3 state HMM to define non-occupied (low), intermediately and highly occupied domains (top). Importantly, the state specific HMM distribution parameters (mean and variance of H3K9me2 enrichment) are very similar between ES cells and neurons (bottom). Note that with a 3 state HMM the percentage of domains in the high state does not change between ES cells and neurons (TN). However, we detect an overall increase of the intermediate state of around 10%. (B) H3K9me2 domains were defined by HMM (see methods). While numbers of domains on chromosome 19 increase from 1618 to 1914 from ES cells to neurons, their domain size distribution is very similar. This was also seen using alternative domain definition methods (data not shown). (TIF) [file pgen.1002090.s004.tif]

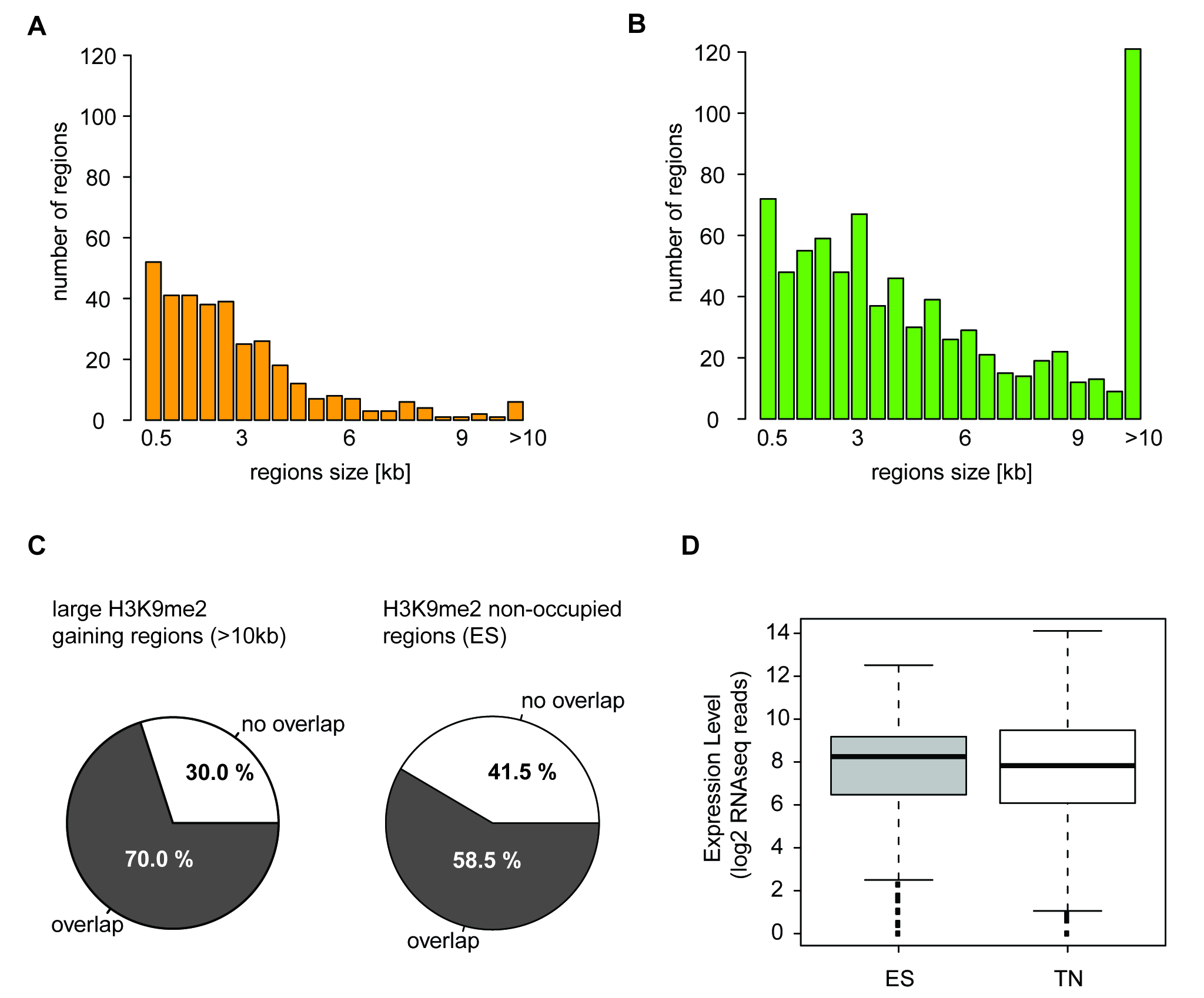

Supplement: Figure S5 — Regions that lose are smaller than regions that gain H3K9me2 during differentiation and gaining regions often overlap with gene bodies. (A and B) Histograms displaying the size distributions of regions that lose (A) and gain (B) H3K9me2 during neuronal differentiation. Adjacent 500 bp windows being in a low HMM state in both ES cell replicates and in a high HMM state in the neuron replicates were grouped into H3K9me2 losing and gaining regions, respectively. (C) Pie chart illustrating the percentage of large (>10kb) H3K9me2 gaining regions within (grey) and outside (white) of genes (left). This overlap is significantly higher than for all regions that have no H3K9me2 in ES cells, i.e. which are in a HMM low state in both replicates (right). (D) Boxplot showing expression levels of all genes (n = 138, adjusted P-value <0.05) that gain H3K9me2 in neurons. Note that the median expression does not change significantly between ES cells and neurons (TN), indicating that there is no global trend towards either up- or down-regulation upon accumulation of H3K9me2 in the gene bodies of these genes. (TIF) [file pgen.1002090.s005.tif]

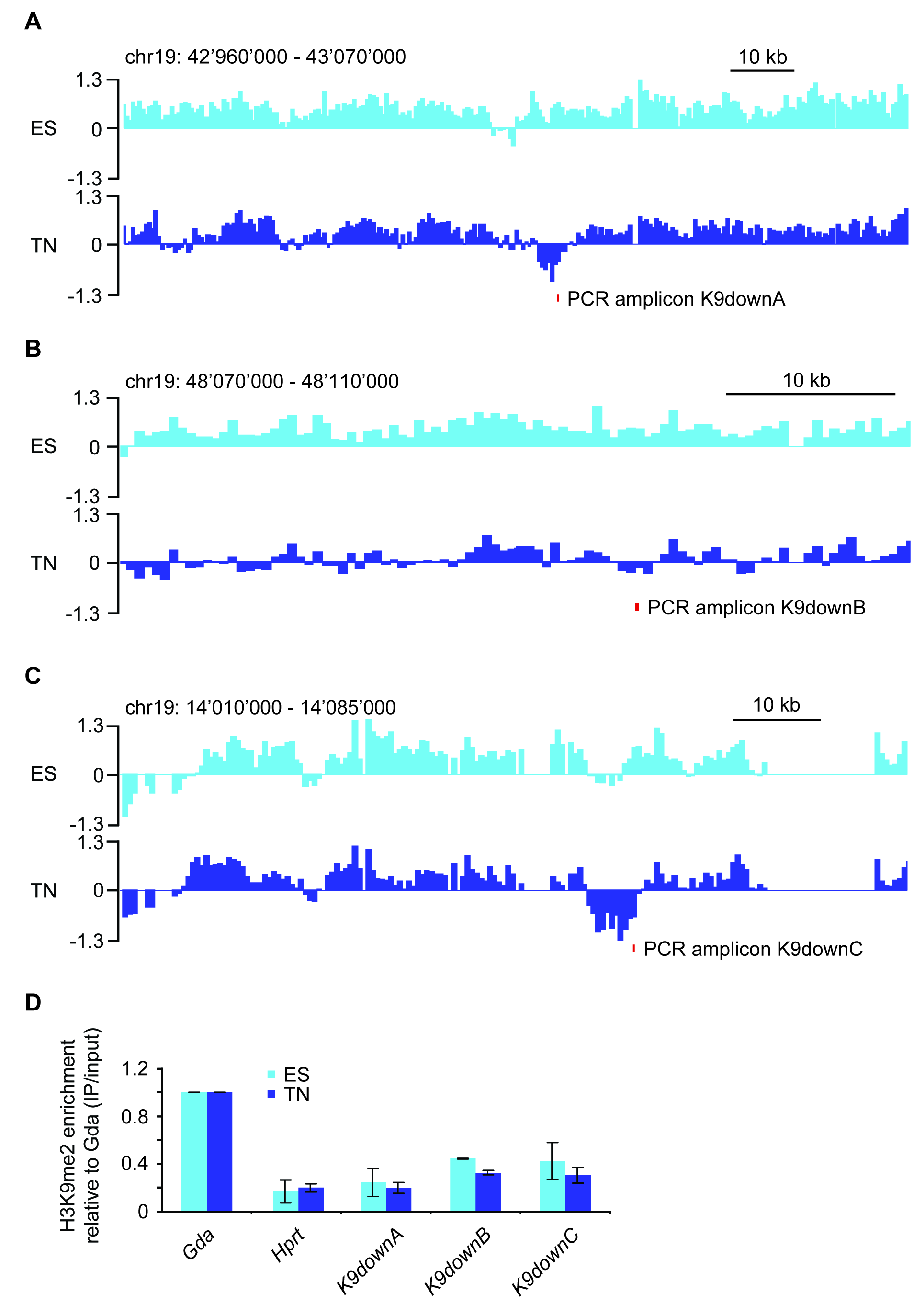

Supplement: Figure S6 — Regions losing H3K9me2 during differentiation are close to background. (A–C) Shown are representative genomic regions that significantly lose H3K9me2 as defined by HMM analysis. (D) ChIP-real time PCR validation of the regions shown in (A–C). Note that H3K9me2 enrichments are already low in ES and only slightly above background as exemplified by the active housekeeping gene Hprt, which does not carry H3K9me2. The loss of enrichment in neurons is small, though reproducible. (TIF) [file pgen.1002090.s006.tif]

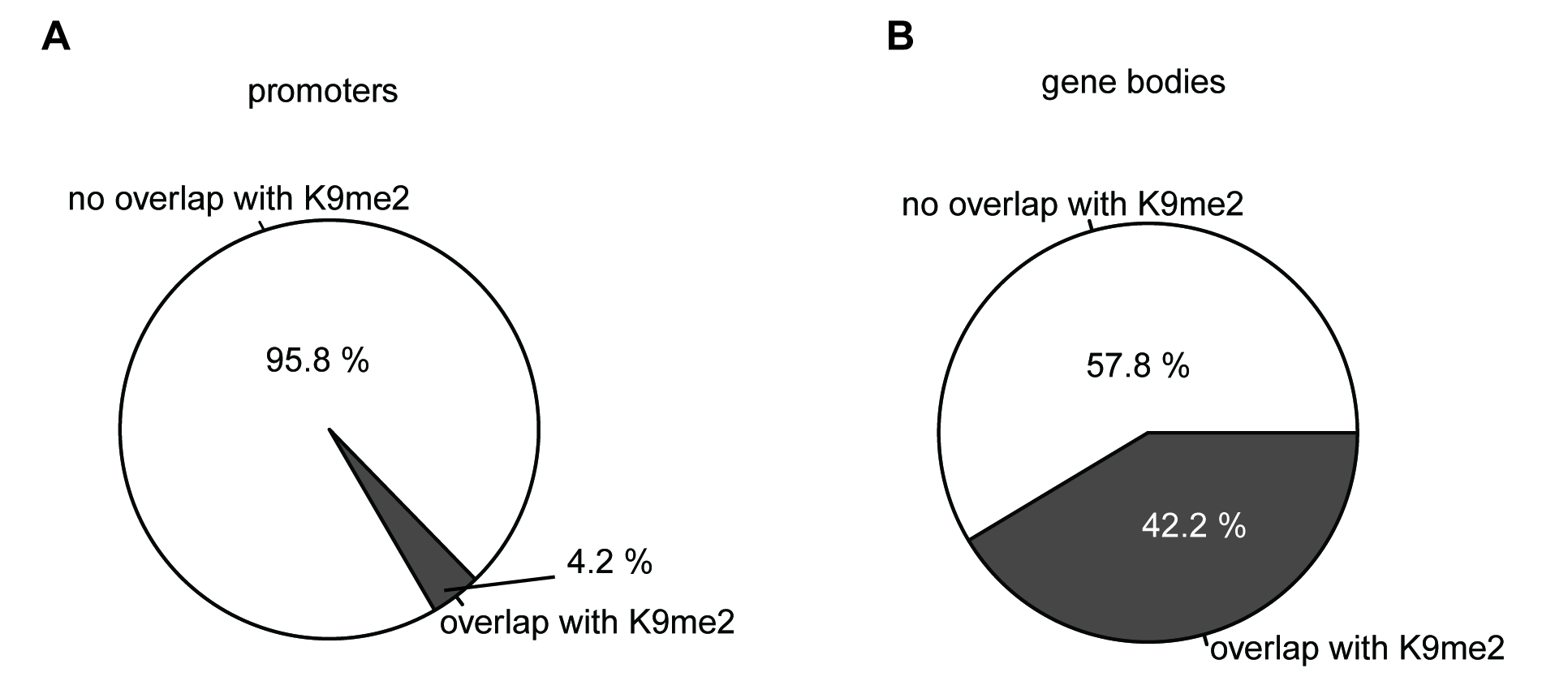

Supplement: Figure S7 — H3K9me2 in neurons rarely overlaps with promoter regions of active genes. (A and B) Pie chart illustrating the percentage of promoters (1 kb up- and downstream of TSS) (A) and gene bodies (B) of transcribed genes that overlap H3K9me2-occupied regions in neurons. Note that promoters are rarely H3K9 dimethylated when the gene is actively transcribed. (TIF) [file pgen.1002090.s007.tif]

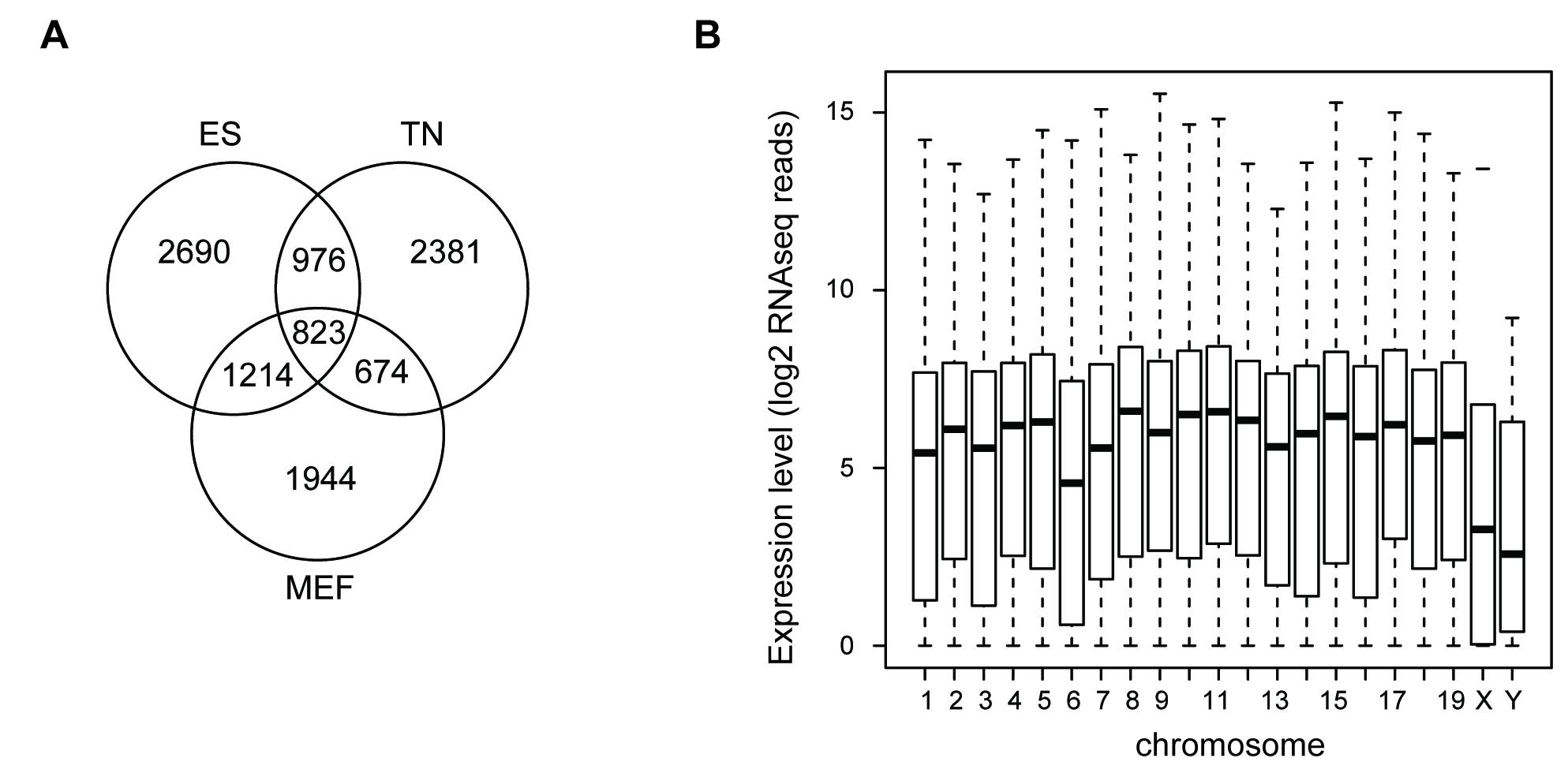

Supplement: Figure S8 — Overlap of low-level expressed genes and expression of transcripts per chromosome. (A) Venn diagram illustrating the overlap of transcripts expressed at a low level (log2 RNAseq reads between 2 and 5) in at least one of the examined cell types. Note that areas are not drawn to scale. (B) Boxplots showing expression levels of transcripts per chromosomes. (TIF) [file pgen.1002090.s008.tif]

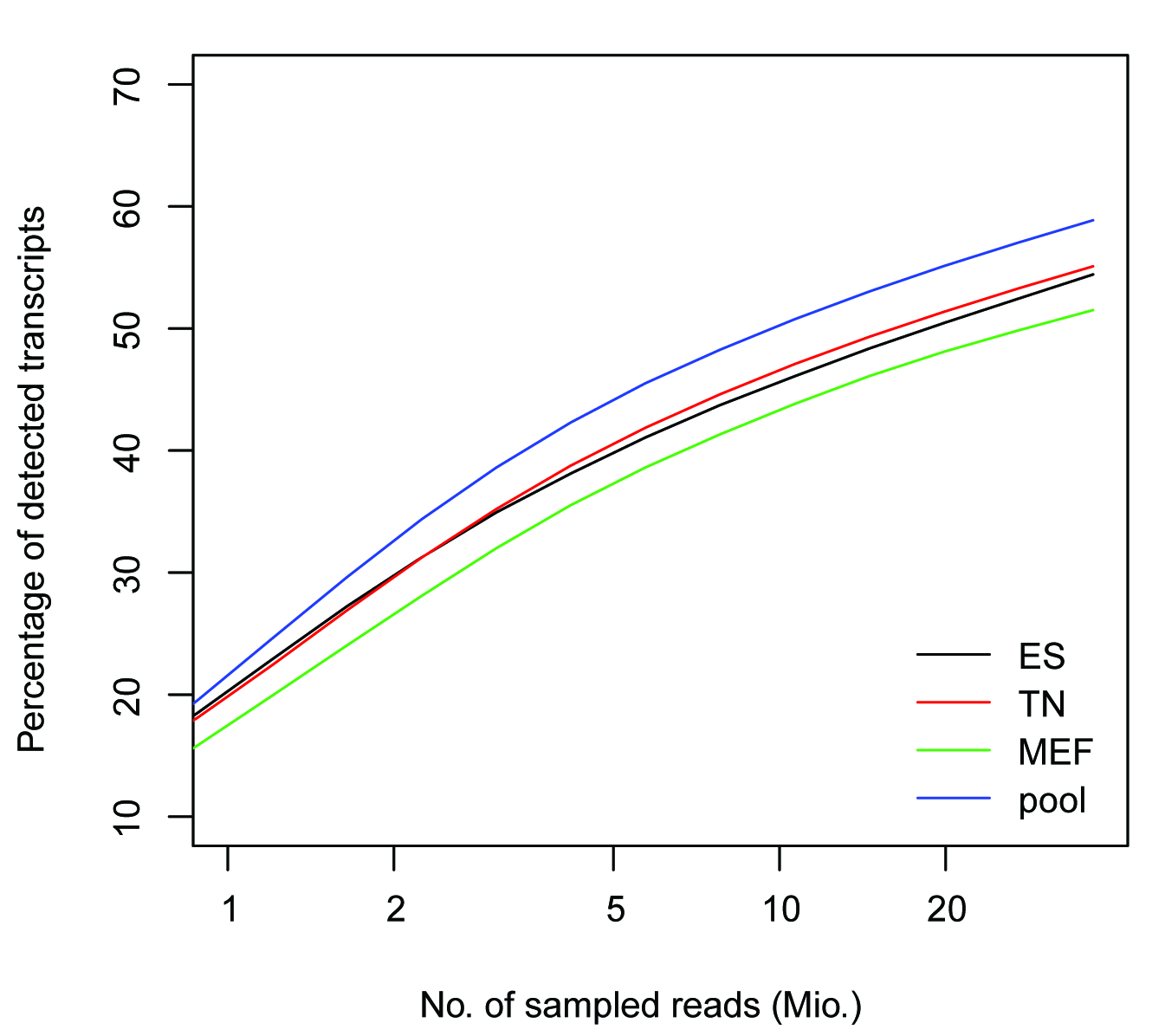

Supplement: Figure S9 — Absence of difference in transcriptome complexity is not a function of sample size in next generation sequencing. Shown is the number of detected Refseq transcripts (at least 32 reads) relative to the number of sampled reads. Number of detected transcripts is shown as the mean of 100 rounds of random subsampling with the same total read numbers. Irrespective of the sample size, a random sampling of RNAseq reads from ES cells (green line), neurons (dark green line) and MEFs (red line) revealed a smaller number of detected transcripts than an artificial sample with pooled reads from all three cell types (blue line) at any given sample size. (TIF) [file pgen.1002090.s009.tif]

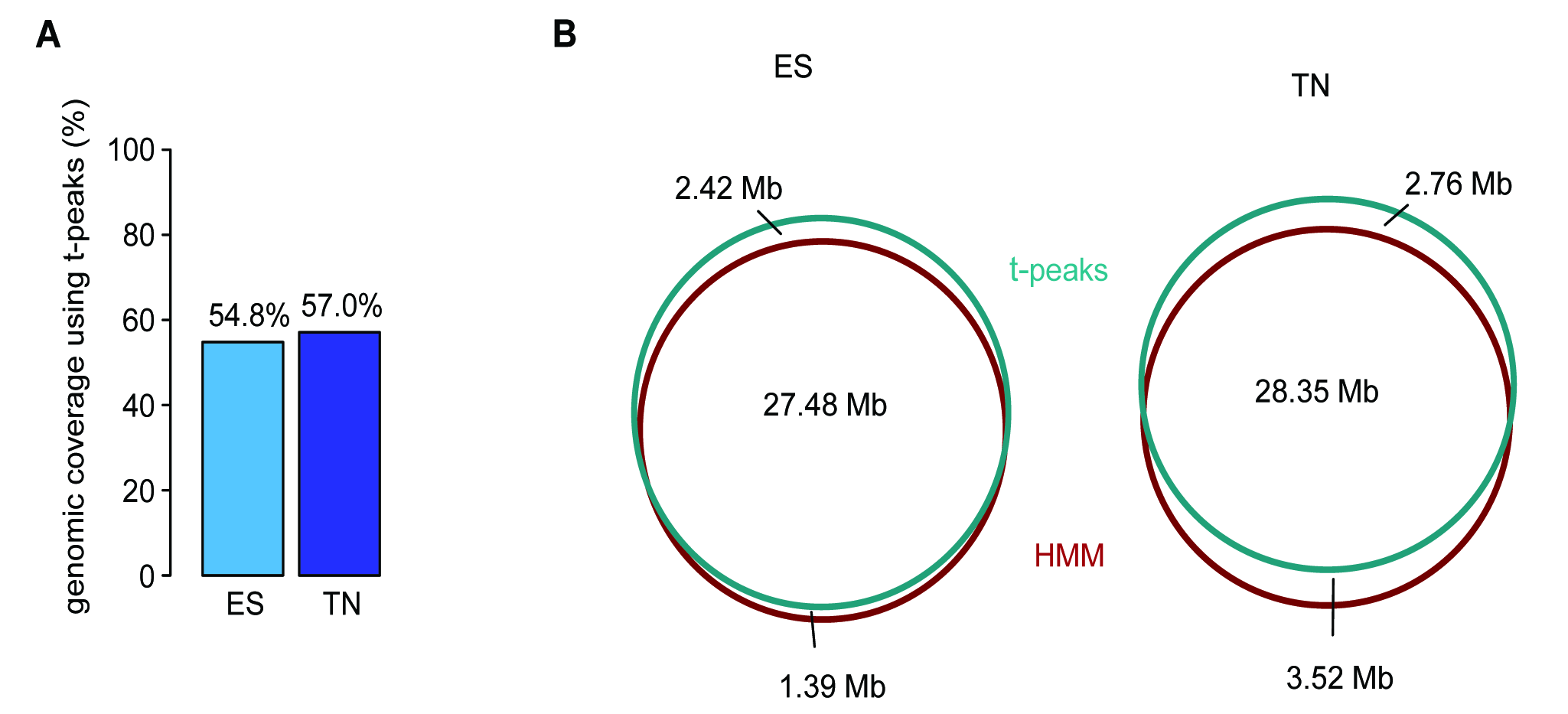

Supplement: Figure S10 — H3K9me2 domain definition by different statistical methods leads to similar results. (A) HMM-independent quantification of genomic coverage of H3K9me2 in ES cells and neurons. Shown is the percentage of chromosome 19 that lies within H3K9me2 peaks defined by a simple threshold method (t-peaks, see Text S1). (B) Diagram illustrating the actual overlap between regions defined to be occupied by H3K9me2 by two different methods; a simple threshold method (t-peaks; see Text S1) and by an HMM approach (HMM; see Text S1). (TIF) [file pgen.1002090.s010.tif]

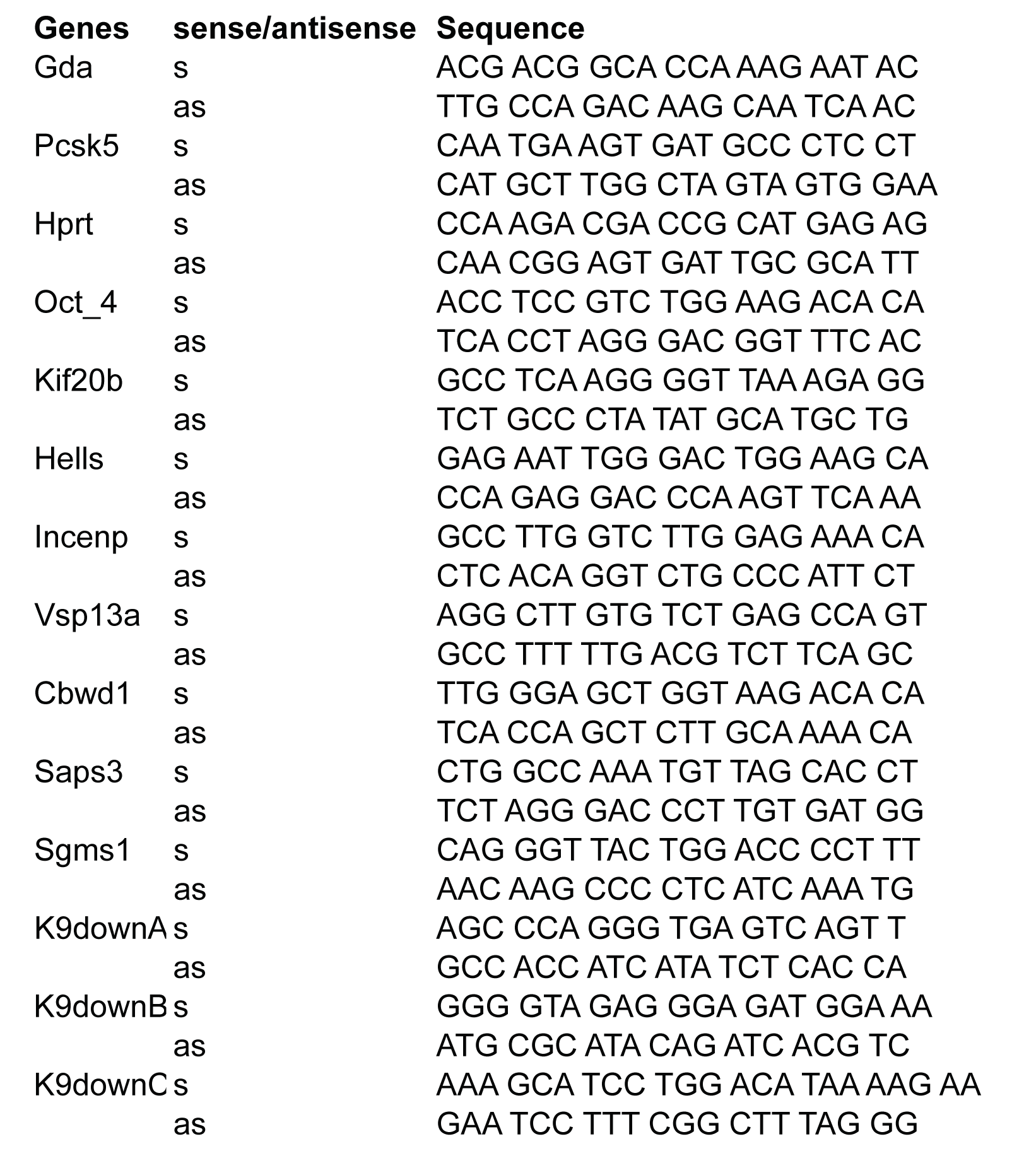

Supplement: Table S1 — List of real time PCR primers. (TIF) [file pgen.1002090.s011.tif]

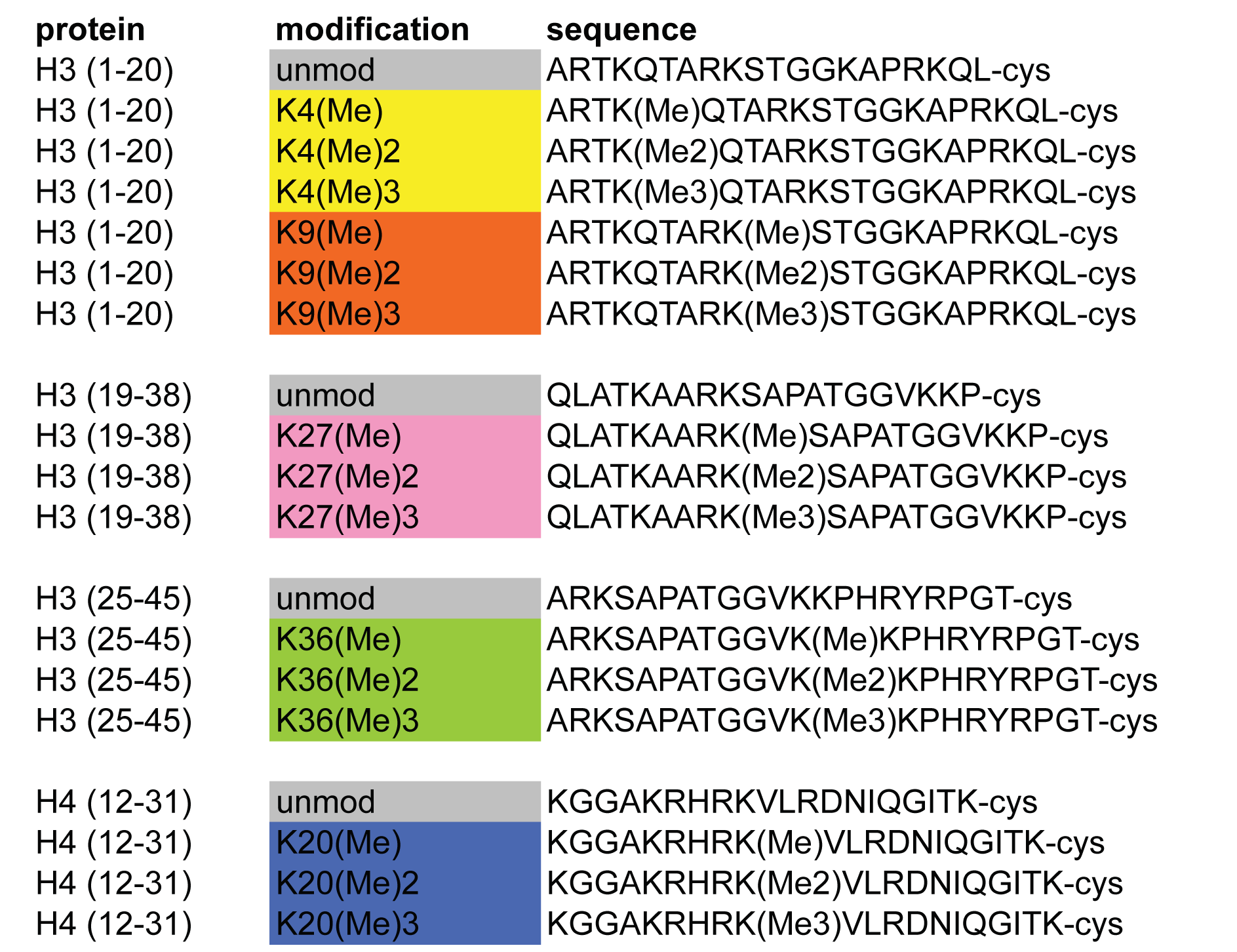

Supplement: Table S2 — Peptides used in dot blots in Figure S1. (TIF) [file pgen.1002090.s012.tif]
