# Supplementary material for: Genomic Prevalence of Heterochromatic H3K9me2 and Transcription Do Not Discriminate Pluripotent from Terminally Differentiated Cells
Source: PLoS Genet. 2011 Jun 2;7(6):e1002090. doi: 10.1371/journal.pgen.1002090 (PMC3107198; doi:10.1371/journal.pgen.1002090)
Supplement: Text S1 — Supplementary methods. (DOC) [file pgen.1002090.s013.doc]

**Supplementary Information**

**Genomic prevalence of heterochromatic H3K9me2 and transcription do not discriminate pluripotent from terminally differentiated cells**

Florian Lienert, Fabio Mohn, Vijay K. Tiwari, Tuncay Baubec, Tim C. Roloff, Dimos Gaidatzis, Michael B. Stadler and Dirk Schübeler

Total supplementary items: 12

**- Figure S1.** Dot blots for assessing the specificity of H3K9me2 antibodies used in this study. Relates to Figure 1 and is discussed in the main text.

**- Figure S2.** Comparison of H3K9me2 profiles with previously published data. Relates to Figure 1.

**- Figure S3.** Comparison of H3K9me2 on chromosome 19 and other chromosome. Is discussed in the main text.

**- Figure S4.** Comparison of HMM analysis parameters. Relates to Figures 1 and 2 and is discussed in the main text.

**- Figure S5.** Analysis of regions that lose or gain H3K9me2 during differentiation. Relates to Figure 2.

**- Figure S6.** Examples of regions that lose H3K9me2 during differentiation. Relates to Figure 2.

**- Figure S7.** Analysis showing that in neurons H3K9me2 rarely overlaps with promoter regions of active genes. Relates to Figure 3.

**- Figure S8.** Overlap of low level expressed genes among cell types and comparison of transcript expression levels among chromosomes. Discussed in main text.

**- Figure S9.** Analysis to validate that the absence of difference in transcriptome complexity is not a function of sample size in next generation sequencing and does not reflect non-saturating sequencing. Relates to Figure 4 and is discussed in main text and Supplementary Methods.

**- Figure S10.** H3K9me2 domain definition by different statistical methods leads to similar results. Discussed in main text and Supplementary Methods.

**- Table S1.** List of real time PCR primers

**- Table S2.** List of peptides used in dot blots for Figure S1

**- Supplementary Methods.** Additional details of the bioinformatical analysis methods

**- Supplementary References**

**Supplementary Methods**

**Segmentation of H3K9me2 profiles by Hidden Markov Models (HMMs)**. To define H3K9me2 enriched regions, we segmented ChIP-chip data using HMMs, as described (Birney et al., 2007). The basic premise of HMMs is that observed data are generated stochastically from a pre-determined number of hidden background probability distributions, or states. We used two states to distinguish enriched from not enriched regions (an analysis using three states led to similar results, see Figure S3). The parameters of the HMMs (emission probabilities, here modeled as normal distributions, and the transition probabilities between states) are estimated via unsupervised learning (Baum-Welch algorithm) from the H3K9me2 enrichment profile. For that purpose, log2 of bound/input ratios were calculated for 500 bp windows on the whole chromosome 19. For single windows not covered by an oligonucleotide on the array the average of the neighboring two windows was taken. Regions consisting of more than one 500 bp window not covered by array were omitted from the analysis. Enriched and not enriched states were assigned to genomic positions according to the most probable path through the trained model states given the observed data (Viterbi algorithm). The analysis was performed independently on each biological replicate and 500 bp windows being in a high state in both replicates were considered to be enriched for H3K9me2. Consecutive genomic positions with identical H3K9me2 enrichment states were merged. For the 3 state HMMs, we performed the analysis in the same way, except for using averaged H3K9me2 enrichment values from the two biological replicates. Analysis was done using the “RHmm” R package (<http://CRAN.R-project.org/package=RHmm> (Taramasco, 2009)).

**Analysis of H3K9me2 changes in gene bodies.** We calculated the average H3K9me2 signal per transcript (RefSeq start to end, downloaded 06/24/2010) as the weighted sum of the log2(ChIP/input) enrichment of oligos within the transcript divided by the weighted number of oligos within the transcript, with oligo weights defined as the fraction of overlap with the particular transcript. Significant changes between ES cells and neurons were then determined using the “LIMMA” R package (Smyth, 2004; Smyth and Speed, 2003).

**Definition of H3K9me2 enriched regions by a simple threshold method (t-peaks).** To cross validate H3K9me2 enriched regions obtained from HMMs by an independent approach we made use of a simple threshold method. For that purpose, average log2 bound/input ratios from two replicates were calculated for 500 bp windows on the whole chromosome 19. For both samples (ES cells and neurons), we defined the respective median of log2 bound/input ratios as a threshold. We then merged adjacent regions with a log2 bound/input ratio above the threshold into domains. Domains separated by 1 kb or less were fused. These H3K9me2 enriched domains show high overlap with domains defined by the HMM approach (Figure S10).

**Deep sequencing analysis.** ChIPseq and RNAseq samples were sequenced on an Illumina GA II analyzer. Low quality reads (not passing Illumina chastity filter or containing more than two N’s) and low complexity reads (based on entropy, typically less than 1%) were removed. We mapped reads to the Mus musculus genome (mm9) and transcriptome (RefSeq, downloaded on 07/17/2009) using bowtie (Langmead et al., 2009) allowing for up to two mismatches and retaining all best hits for reads with up to 100 alignments ignoring sequence qualities.

**Identification of H3K27me3 and H3K4me2 peaks.** H3K4me2 ChIP samples (from one differentiation) and H3K27me3 ChIP samples (from two independent biological replicates) and Input DNA from ES cells (as a background sample) were sequenced on an Illumina Genome Analyzer. Reads were filtered, mapped and counted as described above. We obtained the following number of reads mapping to genome (in Mio. reads); ES_H3K27me3_a1 4.96, ES_H3K27me3_a2 4.60, ES_H3K27me3_b 12.58, ES_H3K27me3_a1 4.59, ES_H3K27me3_a2 4.74, ES_H3K27me3_b 7.5, ES_H3K4me2 13.25, TN_H3K4me2 10.33 and Input 11.9 (with a and b being independent biological replicates and 1 and 2 being technical replicates). In order to identify peaks in the H3K27me3 and H3K4me2 datasets we used MACS peak finder (Zhang et al., 2008). MACS was used with standard settings (lambad-set: 2kb, 10kb, 20kb) and the Input sequencing sample as a background. Peaks were filtered in the following way. We counted the number of reads from the histone modification ChIP library and from the Input library in the peak regions identified by MACS. For each sample we then normalized the number of mapped reads per peak to the total number of reads which could be mapped to the genome. Next, we determined for each peak the enrichment ratio of normalized read numbers from ChIPseq to the normalized reads from Input-seq (after addition of eight pseudo-counts per peak to reduce sampling noise). For further analysis we only used peaks with a two-fold enrichment ratio and more than four mapped reads per 100 bp. We crossvalidated peaks by comparison to peaks identified by two independent methods; BayesPeak (Spyrou et al., 2009) and a variation of the t-peaks method described above. The peaks identified by using these three different methods showed a high overlap (data not shown).

**RNAseq data analysis.** RNA from ES cells, neurons and MEFs from two independent biological replicates was used separately for cDNA preparation followed by sequencing on an Illumina GA II analyzer. Reads were filtered, mapped and counted as described above. We obtained the following number of reads mapping to the transcriptome (in Mio. reads); ES_RNA_a 13.3, ES_RNA_b 35.7, TN_RNA_a 10.8, TN_RNA_b 21.5, MEF_RNA_a 27.2 and MEF_RNA_b 27.5, (with a and b being independent biological replicates). Next, we calculated expression levels of the transcripts as the weighted sum of reads aligning to the transcript, with read weights defined as the observed count divided by the number of genomic hits of each read or the number of hits in a single transcript if greater than the number of genomic hits. This sum was length normalized by dividing through the transcript length and multiplying by the average length of all transcripts. In order to allow comparison among different cell types and biological replicates, transcript levels were normalized to the total number of reads mapping to transcripts for each sequence run. For Repeat analysis, genomic read alignments were compared to the Mus musculus Repeatmasker annotation (Smit, AFA & Green, P, [www.repeatmasker.org](http://www.repeatmasker.org/)) as described for transcriptome analysis. Read counts mapping to 3’927’115 repeat instances were collected in pools according to their corresponding repeat elements and were length-normalized to the summed length of all instances per element. For further analysis we normalized read counts of repeat instances to the total number of reads mapping uniquely to the genome for each sequence run and merged read counts into their corresponding repeat classes.

**Supplementary References**

Birney, E., Stamatoyannopoulos, J.A., Dutta, A., Guigo, R., Gingeras, T.R., Margulies, E.H., Weng, Z., Snyder, M., Dermitzakis, E.T., Thurman, R.E.*, et al.* (2007). Identification and analysis of functional elements in 1% of the human genome by the ENCODE pilot project. Nature *447*, 799-816.

Boyle, E.I., Weng, S., Gollub, J., Jin, H., Botstein, D., Cherry, J.M., and Sherlock, G. (2004). GO::TermFinder--open source software for accessing Gene Ontology information and finding significantly enriched Gene Ontology terms associated with a list of genes. Bioinformatics (Oxford, England) *20*, 3710-3715.

Langmead, B., Trapnell, C., Pop, M., and Salzberg, S.L. (2009). Ultrafast and memory-efficient alignment of short DNA sequences to the human genome. Genome biology *10*, R25.

Smyth, G.K. (2004). Linear models and empirical bayes methods for assessing differential expression in microarray experiments. Statistical applications in genetics and molecular biology *3*, Article3.

Smyth, G.K., and Speed, T. (2003). Normalization of cDNA microarray data. Methods (San Diego, Calif *31*, 265-273.

Spyrou, C., Stark, R., Lynch, A.G., and Tavare, S. (2009). BayesPeak: Bayesian analysis of ChIP-seq data. BMC Bioinformatics *10*, 299.

Taramasco, O. (2009). RHmm: Hidden Markov Models simulations and estimations. R package version 1.3.1.

Zhang, Y., Liu, T., Meyer, C.A., Eeckhoute, J., Johnson, D.S., Bernstein, B.E., Nussbaum, C., Myers, R.M., Brown, M., Li, W.*, et al.* (2008). Model-based analysis of ChIP-Seq (MACS). Genome biology *9*, R137.
